# In-Situ Quantification of the Interfacial Rheological Response of Bacterial Biofilms to Environmental Stimuli

**DOI:** 10.1371/journal.pone.0078524

**Published:** 2013-11-11

**Authors:** Patrick A. Rühs, Lukas Böni, Gerald G. Fuller, R. Fredrik Inglis, Peter Fischer

**Affiliations:** 1 Department of Health Sciences and Technology, ETH Zürich, Zürich, Switzerland; 2 Department of Chemical Engineering, Stanford University, Stanford, California, United States of America; 3 Department of Environmental Sciences and Department of Environmental Microbiology, ETH Zürich and EAWAG, Zürich, Switzerland; Loyola University Medical Center, United States of America

## Abstract

Understanding the numerous factors that can affect biofilm formation and stability remain poorly understood. One of the major limitations is the accurate measurement of biofilm stability and cohesiveness in real-time when exposed to changing environmental conditions. Here we present a novel method to measure biofilm strength: interfacial rheology. By culturing a range of bacterial biofilms on an air-liquid interface we were able to measure their viscoelastic growth profile during and after biofilm formation and subsequently alter growth conditions by adding surfactants or changing the nutrient composition of the growth medium. We found that different bacterial species had unique viscoelastic growth profiles, which was also highly dependent on the growth media used. We also found that we could reduce biofilm formation by the addition of surfactants or changing the pH, thereby altering the viscoelastic properties of the biofilm. Using this technique we were able to monitor changes in viscosity, elasticity and surface tension online, under constant and varying environmental conditions, thereby providing a complementary method to better understand the dynamics of both biofilm formation and dispersal.

## Introduction

Bacterial biofilms, multicellular aggregates of cells attached to surfaces or interfaces that are bound together by an extracellular matrix [1], [2], are considered to be the predominant mode of life of bacteria in nature [3], [4]. This matrix is composed of extracellular polymeric substances (EPS) such as proteins, nucleic acids, amyloid fibrils, and other components secreted by bacterial cells [2]. Biofilm formation is often triggered by changes in the environment and involves several stages often referred to as the biofilm lifecycle. These steps include attachment of the floating cell (s) to a surface, maturation, maintenance and dissolution [5]. Multicellular living offers many advantages for the constituent cells. A principle benefit is protection by the surrounding matrix from environmental stresses such as pH shifts, dessication, UV radiation, and osmotic shock [6], [7].

Bacterial biofilms present a major issue in many medical, industrial, and environmental applications [3]. In food industry, for example, biofilm formation is especially critical as it can lead to food poisoning and outbreaks caused by pathogens such as *Escherichia coli* and *Listeria monocytogenes* [8]–[10]. Although much is known about, the genetics, biochemistry, and biology of biofilms [3], [4], there are relatively few studies examining the physical and mechanical properties of biofilms [11], [12]. Furthermore, very little is known about how these properties change during the course of biofilm development or how they are affected by different environmental variables (e.g. temperature or nutrient availability).

In this study we use interfacial rheology (a technique used to examine the mechanical interfacial stress response to an imposed shear strain) to study how the physical properties of bacterial biofilms vary between different bacterial strains across a range of environmental conditions. Interfacial rheology is often used to study the stability of emulsions and foams, which are stabilized by surface active material. As a consequence, a large variety of systems can be measured by interfacial rheology including proteins [13], [14], surfactants [15], and particles [16] as recently summarized by Sagis and Erni [17], [18]. It has also recently been used to study biofilm formation of a clinically relevant strain of *Escherichia coli* [19]. In order to address the shortcomings of many of the techniques currently used to study the physical properties of biofilm formation (i.e. they often only measure biofilm properties indirectly and struggle to capture real time changes in biofilms structure under fluctuating conditions) we conducted a series of measurements using modified subphase rheometer setup that allows real-time measurement of viscoelastic properties and the ability to alter subphase conditions [20], [21]. We measured the viscoelastic properties of biofilm formation in three bacterial species (*Escherichia coli*, *Pseudomonas fluorescens*, and *Bacillus subtilis*) under a range of different growth conditions. We found that each bacterial species had a unique viscoelastic growth profile and responded differently to changes in the growth medium. We therefore propose that interfacial rheology could be used as a complementary method to better understand biofilm formation.

## Materials and Methods

### Bacterial strains

In comparison to air-solid and liquid-solid biofilms, air-water biofilms are not as commonly studied. However, air-water biofilms, called pellicles, are of increased interest as several pathogenic bacteria can form such pellicles [19]. Recent studies on pellicles have focused on the importance of structural elements in *Pseudomonas fluorescens* [22]–[24] and *Bacillus subtilis* [25]–[29]. Based on this, three biofilm forming bacteria *Escherichia coli*, *Pseudomonas fluorescens*, and *Bacillus subtilis* were chosen and were cultured in either Minimal Salts (M9) glucose (a defined growth medium, which only provides limited nutrients required for bacterial growth) or Lysogeny Broth (LB) (a rich nutrient medium, which contains a range of nutrients). Stock cultures frozen at –70°C in glycerol 30% (v/v) were obtained from various sources. The *E. coli* strain K12 csr (carbon storage regulator gene knock-out) and *P. fluorescens* SBW25 strain were obtained from the Institute of Biogeochemistry and Pollutant Dynamics (ETH Zürich, Switzerland). The *Bacillus subtilis* strain PY79 was obtained from the Institute of Integrative Biology (ETH Zürich, Switzerland). The *Bacillus subtilis* surfactin knockout NCIB 3610 was obtained from Kolter Laboratory (Harvard, USA).

Working cultures were grown from the stock cultures by inoculating Mc-Cartney bottles at 1% (v/v) containing LB broth and incubated at 37°C for 24 h shaking at 160 rpm. Fresh medium was inoculated with 1% (v/v) with this subculture and immediately used for subsequent measurements. All media were prepared with deionized water and sterilised by autoclaving at 120°C for 15 minutes. The components of M9 were autoclaved separately and mixed prior to usage. To see the effect of surfactants after biofilm formation, Tween 20 (Merck, Germany) was used.

### Interfacial rheology

**Figure 1:**
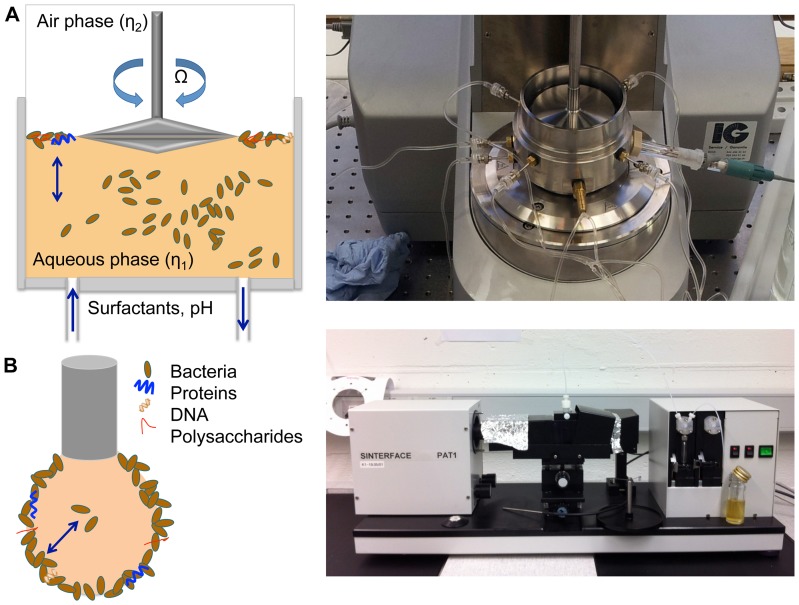
Overview on the experimental techniques used to measure the biofilm elasticity. A: Schematic overview over subphase controlled interfacial rheological setup used for the bacterial biofilm elasticity measurements. B: Schematic representation on the pendant drop tensiometer with an biofilm.



To test the transient build up of the biofilm formation at the water-air interface, a shear rheometer (Phyisca MCR 501, Anton Paar) with a biconical disk geometry was used (see Fig. 1 A). A more detailed methodology is presented in the literature [30]. A short summary of the equations is presented. During interfacial rheological measurements, the interfacial flow is assumed to be decoupled from the bulk phase flow when the Boussinesq number 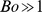 (Bo = 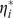/((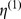
+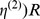)). In this case, the disk rheometer can be treated as a 2D Couette device. The bicone is oscillated at a defined angular velocity 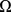 and thus the interfacial shear viscosity 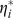 can be calculated as followed:
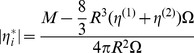
(1)where 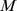 is the torque, 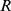 the bob radius of the biconical disk, and 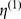, 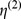 are the viscosities of the two bulk phases. Through a sinusoidal oscillation with a defined deformation 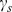
(t) = 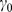  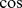
(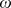
t), a stress response 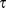
(t) = 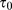  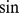
(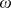 *t+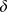
*) with a phase shift 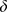 can be measured. With

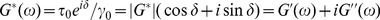
(2)the dynamic complex interfacial shear modulus 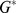
(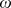) can be calculated. The interfacial storage modulus G′ and the interfacial loss modulus G″ is used to characterize the rheological behavior of the interface. The bacteria were incubated inside a modified measuring cell. During the biofilm build up (t
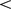
70 h), time sweeps were performed at a constant deformation and frequency (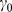 = 0.1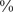 and 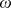 = 1 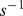) for a general characterization of the biofilm. Unless stated otherwise, measurements were performed at 25°C. In a next step, the subphase was modified through a previously constructed device, which allows simultaneous pH control during interfacial rheological measurements [20]. In short, tubes are connected to the measuring cell, which in turn were connected with syringe pumps. Through in and outlets it was possible to exchange liquid inside of the measuring cell without disturbing the interface. To calculate the Tween 20 concentration 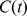 in the subphase, assuming the liquid in the measuring cell is perfectly mixed, the following equation was used:
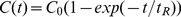
(3)where 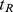 is the residence time of Tween 20 in the measuring cell and 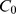 the incoming Tween 20 concentration of the syringe. From the measuring cell volume 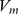 and the volumetric flow rate 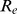, 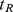 = 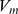/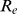 can be calculated [31].

### Pendant drop tensiometry

To measure the surface tension over time of the biofilm formation, a pendant drop tensiometer (PAT-1, Sinterface Technologies, Germany) was used (see Fig. 1 B). A detailed methodology is given in the literature [32]. A drop is formed at the end of a capillary and monitored with a video camera. The Young-Laplace equation is used to fit the resulting drop contour. At a constant drop size, controlled by a piezo element, the transient surface tension 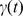 is measured. Measurements were performed at 20°C.

## Results

### Biofilm growth of *E. coli* and *P. fluorescens* in nutrient poor and rich media

**Figure 2:**
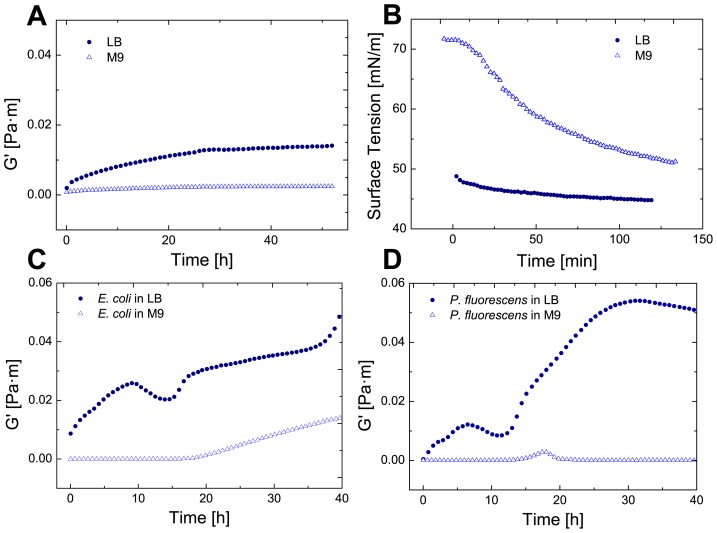
Interfacial elasticity and surface tension of LB and M9 media and bacterial growth in LB and M9 media. A: Interfacial rheology of the pure LB and M9 media at 25°C for 50 hours. B: The surface tension is plotted against the time of pure LB and M9 media. C: The interfacial elasticity is measured of *E. coli* incubated in M9 and LB media. D: The interfacial elasticity is measured of *P. fluorescens* incubated in M9 and LB media.

To investigate the effect of different nutrient levels on biofilm growth of *E. coli* and *P. fluorescens*, a nutrient poor medium (M9 glucose) and a nutrient rich medium (LB) were compared. At first the interfacial storage G' (elasticity) and loss modulus G” (viscosity) of M9 and LB were measured. Adsorption layers, formed by surface active material such as proteins, cause an increase on the elastic and viscous moduli. In our case, both moduli are a function of cell density and network formation. Through direct and indirect interactions between the adsorbed bacteria, the interface becomes viscoelastic. An increase in both moduli is therefore a sign of increased cell adsorption, cell growth at the interface and network formation through the production of biofilm components. The M9 glucose medium does not contain surface components contributing to the film elasticity as observed in Fig. 2 A. However, the LB medium contains proteins and thus displays a viscoelastic protein layer. The surface pressure measurements (see Fig. 2 B) confirm that M9 glucose only contains traces of surface active material whereas LB is saturated with proteins and displays a typical protein adsorption curve (LB medium protein). A constant interfacial elasticity value of LB is reached after 30 hours through rearrangement processes.

In M9 glucose medium only *E. coli* and *P. fluorescens* displayed a visible pellicle after 80 hours, whereas *B. subtilis* cultures were not able to colonize the surface with a film. Consequently no rheological measurements with *B. subtilis* in M9 glucose were performed. The elasticity measurements were performed with both *E.coli* and *P. fluorescens* in both LB and M9 glucose media (see Fig. 2 C and D). As can be observed, both bacteria showed biofilm formation at the interface as the elasticity increased. The first elasticity plateau observable in the elastic growth curves in Fig. 2 C and D represent the typical protein adsorption curve (LB medium protein). The following decrease of elasticity is presumably caused by metabolic reactions by the bacteria. Due to the bacterial metabolism, glucose present in the medium is used up and the resulting acidification causes a decrease of elasticity (as discussed later). After t = 15–20 h, the biofilm elasticity increased in strength. As the biofilms formed in M9 glucose medium only show low level (*E.coli*, see Fig. 2 C) or levels of elasticity close to the measuring limit (*P. fluorescens*, see Fig. 2 D), we chose to continue measuring in LB medium as the biofilms formed in LB display Boussinesq numbers higher than 1.

**Figure 3:**
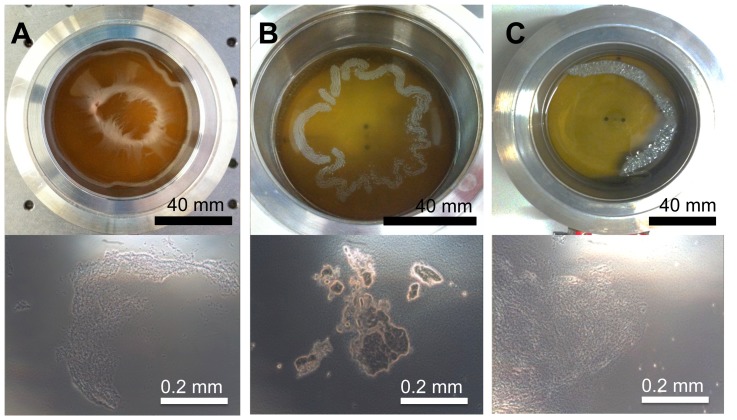
Biofilm formation at the water-air interface. Macroscopic (top) and microscopic images (bottom) of biofilms formed at the water-air interface after 72 h of *P. fluorescens* (A), *E. coli* (B) and *B. subtilis* (C).

**Figure 4:**
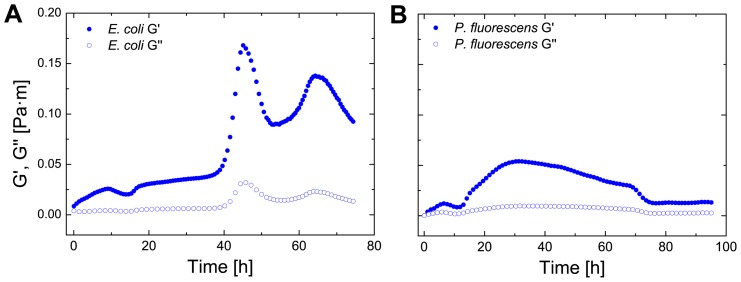
Transient biofilm elasticity of *E. coli* and *P. uorescens*. The elastic (G0) and viscous (G00) as a function of time for *E. coli* (A) and *P. fluorescens* (B).

To confirm our rheological measurements, we observed the biofilm formation in LB both macroscopically and microscopically with a light microscope (Fig. 3). All three observed bacteria formed biofilms of different morphology and structure after 72 hours. *P. fluorescens* biofilms had a slimy texture (Fig. 3 A), *E. coli* formed a brittle network (Fig. 3 B) and *B. subtilis* formed a thick layer (Fig. 3 C). Under the microscope, a 3 dimensional biofilm structure was observed for all bacteria. To observe the interfacial elasticity changes caused by the three bacteria, the three bacteria were grown in LB medium for up to 80 hours. In Fig. 4 both interfacial moduli of *E. coli* and *P. fluorescens* are plotted from t = 0 h to t = 80 h. As can be observed, the elastic moduli G' is dominant, thus the biofilm is predominantly elastic. In Fig. 4 B it is visible that the elasticity decreases after reaching a plateau. A similar dynamic elastic behavior can be observed for Fig. 4 A. Here the elasticity rises sharply after a time of 42 h and decreases rapidly afterwards. A second peak is reached after 70 hours. Both graphs show the dynamic behavior of biofilms, which in comparison to protein adsorption layers, show a varying elasticity over time. As the elastic moduli are higher than the viscous moduli only the storage modulus G' is plotted in future graphs.

### Temperature effect on biofilm elasticity of *E. coli* and *P. fluorescens*

**Figure 5:**
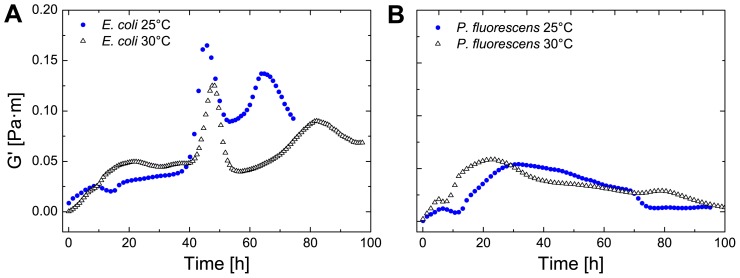
Effect of temperature on biofilm formation of *E. coli* and *P. fluorescens*. The elastic (G0) and viscous (G00) is plotted against the time of *E. coli* (A) and *P. fluorescens* (B) with changing temperature from 25°–30°C.

To investigate the effect of the bacterial growth on the biofilm elasticity, the temperature was raised to 30°C (see Fig. 5). As expected, with an increased temperature to the optimum of the bacterial growth, the elasticity rose. The effect is twofold, as the elasticity of protein assemblies at higher temperature decreases while on the contrary the optimum of the bacterial growth curve is reached. For both temperatures (25 and 30°C) the curve progressions are very similar which shows that each bacterium possesses a characteristic transient elastic curve. *P. fluorescens* showed a shift on the time scale to shorter times. At 30°C the bacterial metabolism is accelerated as this temperature is closer to the optimum growth temperature. Consequently, bacterial growth and biofilm formation starts earlier.

### Biofilm growth of *B. subtilis* in LB

**Figure 6:**
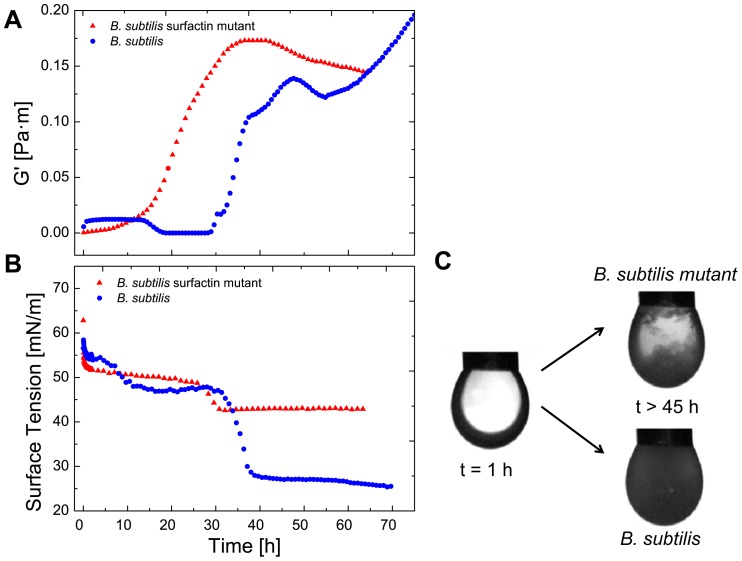
Effect of surfactin production on biofilm formation of *B. subtilis*. A: The elasticity (G0) of *B. subtilis* and *B. subtilis* surfactin mutant is plotted against the time. B: The surface tension versus time is plotted of *B. subtilis* and *B. subtilis* surfactin mutant. C: Images of the pendant drop experiment of *B. subtilis* and *B. subtilis* before and after biofilm growth (C) (t>45 h).

Using interfacial rheological measurements, we were also able to detect subtle changes in biofilm formation caused by single, excreted gene products, such as surfactin. In Fig. 6 A the transient evolution of the elasticity for *B. subtilis* and a surfactin knockout mutant *B. subtilis* is depicted. Both strains we chose are able to form biofilms. In the first 15 hours the protein adsorption is observed for both bacteria. After t>15 h a sharp decrease of elasticity could be observed for the *B. subtilis* strain. The mutant strain, which lacks the surfactin gene sfrA-A, showed no decrease in elasticity in the same observed time frame, as it can no longer produce surfactin, a biosurfactant. Through the replacement of proteins by surfactants, the surface tension is decreased. Surface tension measurements were performed to observe the surface tension development over time of the *B. subtilis* and the mutant *B. subtilis* strain (Fig. 6 B). The first plateau of surface tension is reached after 20 hours. This is created by proteins and the typical surface tension values correspond to the adsorption of proteins. After t>20 hours the surface tension is lowered by the production of surfactin. The strong decrease was not observed for the corresponding mutant strain. A slight decrease is observed, as bacteria possess a varying number of small molecules, which have an amphiphilic character. Biofilms formed without the help of surfactin did not completely cover most of the surface as observed in Fig. 6 C.

### Bacterial biofilm interfacial elasticity with modified subphase conditions

**Figure 7:**
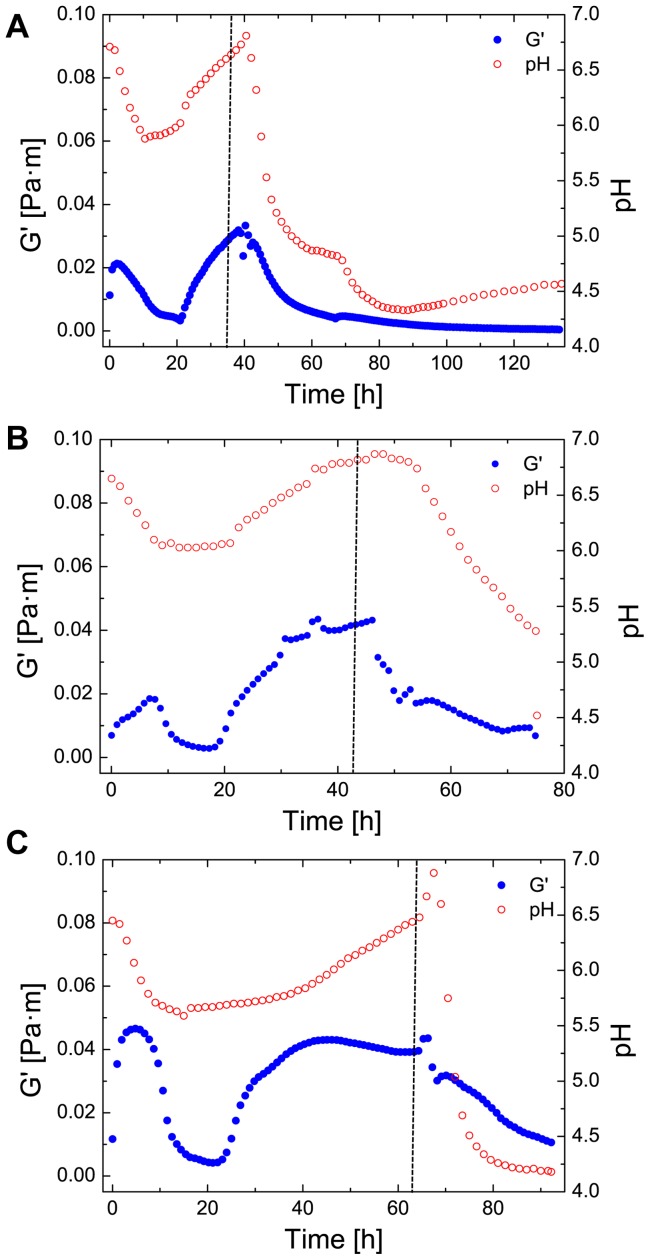
Effect of varying pH on biofilm elasticity after biofilm formation. The elasticity (G0) is plotted against the time of *E. coli* (A), *P. fluorescens* (B) and *B. subtilis* (C) before and after pH change. After the dotted line, the pH was controlled by the addition of 1 M HCl.

Cleaning agents for industrial removal of biofilms often contain combinations of surfactants, disinfectants and possess a low pH [33]. The pH has a strong effect on the physico-chemical properties of proteins and on bacterial growth and metabolism. To investigate pellicle behavior at a certain pH, hydrochloric acid (HCl) at a concentration of 0.25 M was injected into the subphase. This allowed us to simulate a changing environmental condition after bacterial biofilm formation (see Fig. 7). In a first step, the biofilm was allowed to grow under constant subphase conditions (t
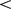
30 h). After changing the pH, for all bacteria a strong dependency between pH and elasticity was observed. After an initial rise of elasticity due to protein and bacterial adsorption (t = 10–15 h) the networks display a decrease of elasticity, which can be accounted through the natural acidication of the medium through the production of small amounts of acids as metabolic by-products. The subsequent rise in pH and elasticity originates from proteolysis of the LB medium and bacterial adsorption [34]. Additionally, the increasing pH leads to an increase of attractive forces, which in turn increases the biofilm elasticity. After biofilm formation, the pH was lowered to a pH 4–5. The pH lowering led to a decrease of elasticity of the biofilm. A strong dependency of pH on network elasticity of amyloid fibrils at water-oil interfaces was observed in a recent study [20]. Eventually, the pH was so low, that no elasticity was detected for all analyzed biofilms.

**Figure 8:**
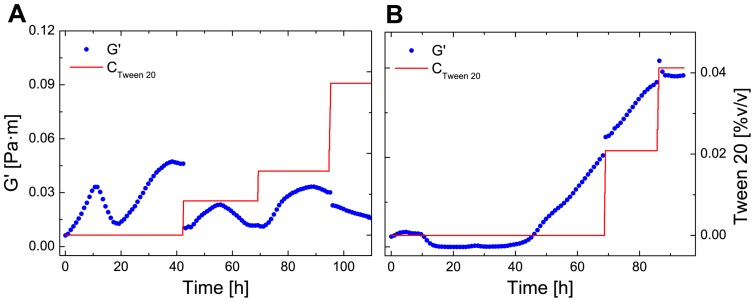
Effect of Tween 20 on biofilm elasticity after biofilm formation. The elasticity (G0) is plotted against the time and concentration of Tween 20 of *P. fluorescens* (A) and *B. flsubtilis* (B).

Besides pH and mechanical treatments, biocides and disinfectants are of high importance to control and remove biofilms in industry [35]. They combine a bactericidal or bacteriostatic effect and remove proteins and polysaccharides. Cells in biofilms are much less susceptible to these chemical treatments than planktonic cells as they are protected by the surrounding matrix [7]. To determine the effect of surfactants on mature biofilms, Tween 20– a non-ionic polysorbate surfactant – was added to the subphase. Surfactants are small molecules that adsorb to the interface very quickly. Given their small size, they are able to intercalate in an existing protein network. The effect is twofold, as they can solubilize protein into the subphase and displace protein from the interface as they lower the interfacial tension more efficiently [36]–[38]. In most protein-surfactants competitive studies, the result of added surfactant is a loss of elasticity [39]. The surfactant was added in several applications to both *P. fluorescens* and *B. subtilis* biofilms at a concentration of 1%(v/v). Through equation 3 the concentration of Tween 20 in the measuring cell was calculated. In Fig. 8 A the elasticity of the *P. fluorescens* biofilm is shown after the addition of Tween 20. At first there is a sharp decrease after the addition of Tween 20. The elasticity of the *P. fluorescens* biofilm recovers after the first application and is not strongly affected by subsequent additions. In comparison to *P. fluorescens*, the elasticity of the *B. subtilis* biofilm was not affected after injection of Tween 20 as depicted in Fig. 8 B.

## Discussion

In this study we used interfacial rheology to monitor bacterial biofilm formation at the air-liquid interface. In particular we used interfacial rheology to measure the elasticity and viscosity of the system, which provided information about biofilm cell density increase and connectivity between bacterial cells, whereas we used pendant drop measurements to yield information about the surface tension development over time. Cultures of the three bacterial strains *E. coli*, *P. fluorescens* and *B. subtilis* were inoculated under a range of different environmental conditions and subsequent biofilm formation was measured. Our interfacial rheological setup also allowed us to simulate and measure the effects of various environmental stresses such as changing temperature, pH, and surfactants on biofilms.

We found that nutrient levels had large effects on the rheological characteristics of biofilm formation. For example when *E. coli* and *P. fluorescens* were grown in a low nutrient medium (M9 glucose), the resulting biofilms were much weaker and showed a different formation profile when compared to a high nutrient medium (LB broth). There was also substantial variation between the rheological properties of all three bacterial strains with a characteristic and dynamic growth profile for each strain (see Fig. 4 and Fig. 6 A). No plateaus of elasticity were observed. This can be explained by the biofilm lifecycle [3]. As the bacteria start to starve, as new medium is not introduced, the biofilm is recycled for nutrients and bacteria leave the biofilm. The elasticity decrease observed for *E. coli* and *P. fluorescens* however never decreased completely inside the observed time frame as most proteins are irreversibly adsorbed at the interface. In comparison to protein adsorption layers, bacterial biofilms displayed a much more dynamic behavior with varying elasticity over time (see Fig. 4 and Fig. 6 A).

We also measured the effect of temperature shifts on the elasticity profile of the bacterial biofilm. Although, there were slight changes in the speed of biofilm formation, the qualitative elasticity profile remained largely the same. An increased temperature close to the optimum growth temperature of the bacterium should lead to an increase in elasticity. However, protein connectivity in interfacial adsorption layers is also influenced by temperature, thus the effects of temperature on biofilm growth can have positive and negative effects. Although the differences caused by the increase in temperature are therefore not significant for each bacterium, we still see substantial variation between the bacterial elastic profiles of *E. coli* and *P. fluorescens*.

Our system was also able to detect small differences in biofilm formation due to differential gene expression. By culturing wild-type strain of *B. subtilis* and comparing this to surfactin knockout strain (deficient in the surfactin gene sfrA-A), we were able to observe differences in biofilm formation. Whereas the *B. subtilis* wildtype strain displays a sharp decrease of elasticity is due to the production of surfactin (a biosurfactant, which can have antibacterial effects), the knockout mutant no longer exhibits this characteristic drop in elasticity [26], [40], [41]. Under normal biofilm forming conditions *B. subtilis* uses this surfactant to spread on the water-air interface by lowering the surface tension. Microcolonies of *B. subtilis* produce surfactin, leading to surface tension gradients, which promote cooperative spreading of the cells, and is important for dispersal in environments with no external fluid flows. This enhanced spreading through cooperative motility promotes biofilm formation and allows the cells to spread over or between nutrient sources [42]. Using interfacial rheology, we were able to observe the progression of surfactin production, colonization of the liquid-air layer by bacterial cells, and the subsequent biofilm formation. Although a slight decrease in elasticity can be observed in our mutant strain, this can be attributed to the production of a varying number of small molecules, which have an amphiphilic character. As the mutant lacks the production of surfactin, it was not able to colonize the surface as homogenously as the wild-type *B. subtilis* (Fig. 6 C), and provides an example of how the changes in gene expression can influence physical properties of the pellicle and thus can be monitored by using interfacial rheology.

Finally, to observe the influence of changing environmental conditions, we modified the subphase by adding a strong acid and an artificial surfactant. When hydrochloric acid was added, we found that pH and elasticity are strongly dependent on each other in the observed biofilm networks. With pH values around 4 the biofilm network was completely disintegrated, which implies that the network strength of the matrix is strongly affected by pH. This has also been observed for networks composed of amyloid fibrils adsorbed at the water-oil interface, as a decrease in pH causes a decrease of attractive forces [20]. Many bacterial biofilms are believed to contain amyloid fibrils as structural elements and are widely present in natural biofilms [43]. They occur in *E. coli* as fimbriae [19], [44], in *B. subtilis* [29] and in *P. fluorescens* [45]. As the pH decrease leads to zero elasticity, the leading structural element are the amyloid fibrils. Thus, if the bacteria loose their ability to form amyloid fibrils, only a weak biofilm formation at the water-air interface should be observed. However, other biofilm matrix components are also likely to be affected by pH, as well as bacterial metabolism and growth, which can lead to changes in the biofilm elasticity. To study the effect of surfactants on formed biofilms, Tween 20 was injected to the subphase after biofilm formation. Contrary to expectations, the elasticity of *P. fluorescens* biofilm weakened initially but then showed a recovery effect of elasticity. The biofilm seems to show a complicated elasticity curve response to the several additions of Tween 20. Several reasons might cause this changing elasticity over time. The elasticity did not vanish completely which may be due to the thickness of the pellicle. Furthermore the network contains other macromolecules that are entangled, preventing it from being removed from the interface. The elasticity of the *B. subtilis* biofilm was not affected after injection as depicted in Fig. 8 B. Biofilms formed by *B. subtilis* form very ordered structures and thus no free standing interface is available for the intercalation of Tween 20, as it is known that surfactants use gaps in the interfacial adsorption layer as nucleation points for additional surfactant adsorption [36].

Biofilm formation is a complex process where a variety of structural elements change over time. We propose that with interfacial rheology the different stages of biofilm growth can be identified and measured. First, the initial attachment by the bacteria can be registered through interfacial rheology and pendant drop tensiometry. After the initial bacterial attachment, the biofilm grows and forms a complex viscoelastic material. The online measurements show that biofilm formation is not necessarily just simple growth, but may contain several different biofilm stages, as reflected by the observed changes in elasticity over time. In bioassays, these different stages are often missed. Identifying the different stages should help to understand the development of biofilm formation and find the optimal conditions for biofilm removal. Biofilm cohesive strength, reflected by the elastic and viscous moduli, is a very important characteristic of biofilms as its quantification over time allows a deeper understanding of biofilm formation and detachment. One of the several advantages of measuring the elasticity and viscosity through interfacial rheology, is that in comparison with particle tracking, the whole biofilm is measured. This avoids problems which might arise due to the heterogeneous nature of biofilms. Additionally, interfacial rheology in oscillatory mode (used in this study) provides a method, which is minimally invasive and online, so a quantitative observation over time without destroying the biofilm is possible. By using the modified setup, the direct influence of changing environmental conditions is possible thus allowing for example to observe a variety of molecules (e.g. antibiotics) and their effect on biofilm strength. Additionally, mutations in biofilm forming genes can be observed through changes in the elasticity profile. We were also able to show that the transient elasticity behavior is highly dependent on bacterial type and media, thus providing a physical quantitative value which can be attributed to each bacteria and not only a percentage of biofilm growth. Further analysis through large amplitude oscillatory shear experiments could provide more in depth information about the force needed to disrupt the biofilms. The results show, that interfacial rheology proved to be an effective method to measure biofilm formation online at the air-liquid interface. In combination with pendant drop measurements, these methods help to better understand the complex matter of biofilms and gain further insights in their still poorly understood mechanical properties.
